# Suicide and self-harming behaviour in Europe: Current trends and prospects

**DOI:** 10.1192/j.eurpsy.2025.70

**Published:** 2025-08-26

**Authors:** P. A. Saiz

**Affiliations:** 1 Departmen of Psychiatry, University of Oviedo; 2 CIBERSAM; 3 ISPA; 4 SESPA, Oviedo, Spain

## Abstract

**Abstract:**

Suicide and self-harming behaviors represent significant public health challenges across Europe, with profound implications for individuals, families, and communities. This presentation aims to explore current trends in suicide and self-harm, highlighting variations across different European countries and demographic groups. Recent data indicate a concerning rise in self-harming behaviors, particularly among adolescents and young adults, often linked to factors such as mental health disorders, social isolation, and the impact of the COVID-19 pandemic. Furthermore, the stigma surrounding mental health issues continues to hinder open discussions and access to care, exacerbating the problem.Looking ahead, this presentation will propose future directions for research and practice, advocating for a multidisciplinary approach that integrates psychological, social, and medical perspectives. By fostering collaboration among healthcare providers, policymakers, and community organizations, we can enhance our understanding of suicide and self-harm, ultimately leading to more effective prevention and intervention strategies. This session aims to stimulate dialogue and inspire innovative solutions to address these pressing issues in Europe, promoting mental well-being and reducing the incidence of suicide and self-harming behaviors.

**Disclosure of Interest:**

P. Saiz Grant / Research support from: “laCaixa” Foundation, Government of the Principality of Asturias, Instituto de Salud Carlos III, Consultant of: Angelini Pharma, Ethypharm Digital Therapy, Johnson & Johnson, Rovi, and Viatris España.

